# The effects of Aminophylline on clinical recovery and bispectral index in patients anesthetized with total intravenous anaesthesia

**DOI:** 10.12669/pjms.306.5853

**Published:** 2014

**Authors:** Sina Ghaffaripour, Mohammad Bagher Khosravi, Ashkan Rahimi, Mohammad Ali Sahmedini, Abdolhamid Chohedri, Hilda Mahmoudi, Mohammad Reza Kazemi

**Affiliations:** 1 Sina Ghaffaripour, Associate Professor, Shiraz Anesthesiology and Critical Care Research Center, Department of Anesthesiology, Shiraz University of Medical Sciences, Shiraz, Iran.; 2 Mohammad Bagher Khosravi, Associate Professor, Shiraz Anesthesiology and Critical Care Research Center, Department of Anesthesiology, Shiraz University of Medical Sciences, Shiraz, Iran.; 3 Ashkan Rahimi, Anesthesiologist, Shiraz Anesthesiology and Critical Care Research Center, Department of Anesthesiology, Shiraz University of Medical Sciences, Shiraz, Iran.; 4 Mohammad Ali Sahmedini, Associate Professor, Shiraz Anesthesiology and Critical Care Research Center, Department of Anesthesiology, Shiraz University of Medical Sciences, Shiraz, Iran.; 5 Abdolhamid Chohedri, Associate Professor, Shiraz Anesthesiology and Critical Care Research Center, Department of Anesthesiology, Shiraz University of Medical Sciences, Shiraz, Iran.; 6 Hilda Mahmoudi, Community Medicine and Public Health Specialist. Shiraz Anesthesiology and Critical Care Research Center, Department of Anesthesiology, Shiraz University of Medical Sciences, Shiraz, Iran.; 7 Mohammad Reza Kazemi, Anesthesiologist, Shiraz Anesthesiology and Critical Care Research Center, Department of Anesthesiology, Shiraz University of Medical Sciences, Shiraz, Iran.

**Keywords:** Aminophylline, Bispectral index, Recovery, TIVA

## Abstract

***Objective: ***Aminophylline, which is clinically used as a bronchodilator, antagonizes the action of adenosine, so it can be used to shorten the recovery time after general anesthesia. Therefore, we wanted to test the hypothesis that the administration of aminophylline leads to an increase in bispectral index (BIS) and clinical recovery in patients anesthetized with total intravenous anesthesia (TIVA).

***Methods***: Ninety two patients who were scheduled for elective inguinal herniorrhaphy were enrolled in this study. All patients were premedicated with midazolam and morphine. Anesthesia was induced with propofol 2.5 mg /kg and remifentanil 2.5 µg/kg without muscle relaxant. For maintenance of anesthesia we used propofol 100µg/kg/min, remifentanil 0.2µg/kg/min and 100% oxygen with stable BIS readings in the range 40-60. After skin closure, aminophylline 4mg/ kg was given to Group A and an equivalent volume of normal saline to Group P. BIS values, heart rate, blood pressure, oxygen saturation and End tidal CO2(ETco2) were determined. Time to eye opening, extubation time and response to command were measured.

***Results*** **: **There were no significant differences in SpO2, ETco2 and anesthesia time. Heart rate and systolic blood pressure were found to be statistically higher (p<0.001) in Group A. Time to eye opening, hand grip and extubation were significantly shorter (p<0.001) in Group A. Bispectral index scores were significantly higher in group A.

***Conclusions: ***Injection of aminophylline at emergence time led to significant increase in BIS and shortening recovery time from anesthesia.

## INTRODUCTION

The main role of bispectral index (BIS), which was developed from a processed electroencephalogram, is measuring depth of anesthesia and adjusting the dosage of sedating medications.^1^^,^^2^ The bispectral index ranges from 0 to 100. It is believed that there is a relationship between the BIS and responsiveness. A score of 95 to 100 correlates with an awake state and zero shows no EEG activity. During general anesthesia, a BIS index of less than 55 is recommended.^3^^-^^5^ Intense surgical stimulation increases the BIS and also heart rate and blood pressure.^6^

Aminophylline is a compound of theophylline with ethylenediamine and most common uses in the airway obstruction such as asthma and COPD.^7^^,^^8^ However, emprical evidence suggests that aminophylline, as an adenosine antagonist, can improve the recovery time from general anesthesia.^9^^-^^12^ Adenosine is present in all cells and its receptors are distributed in all brain cells.^13^^,^^14^ Infusion of adenosine in low doses can potentiate the hypnotic effects of anesthetics. Conversely, administration of aminopylline produces some degree of resistance to anesthesia.^15^ Aminophylline can reverse the effect of anesthetics such as benzodiazepines, barbiturates and volatile anesthetics.^16^^-^^18^ The aim of the present study was to investigate the effects of aminophylline on BIS as well as its clinical use in anesthesia recovery from TIVA.

## METHODS

After obtaining ethical committee approval and written informed consent, 92 adult male and female patients between ages 18-50 years old and American Society of Anesthesiologists (ASA) physical status I and II, who were scheduled for elective herniorrhaphy with a duration of less than one hour were enrolled in this study. The study was designed as a double-blind, randomized, controlled trial and the patients were divided in two groups by simple randomization. Exclusion criteria were: hypersensitivity to aminophylline or other methylxanthines, egg and soy bean; history of opioid addiction, consumption of sedative-hypnotic or psychoactive drugs in last one year, chronic therapy with aminophylline or other methylxanthines and positive history of cardiac arrhythmia, palpitation and convulsion. On arrival in the operating room, ECG, non-invasive arterial pressure, heart rate, ETco2 and SpO2 were monitored. The BIS monitoring electrode was placed on the patient’s forehead after careful cleaning of the skin and was continuously recorded from a bifrontalmontage using the aspect EEG monitor. All patients were premedicated with 0.05mg/kg of midazolam and 0.1mg/kg of morphine. Anesthesia was induced with propofol 2.5mg/kg, and remifentanil 2.5 µg/kg. Without any muscle relaxant tracheal intubation was done. Anesthesia was maintained with propofol 100µg/kg/min, remifentanil 0.2µg/kg/min and 100% oxygen with stable BIS readings in the range 40-60. At the end of surgery, after skin closure and discontinuing TIVA, the study drug was injected. The syringes were prepared by an independent anesthesiologist and contained either aminophylline or equivalent volume of normal saline. Patients were received saline or aminophylline 4mg/kg within two min. Recovery was assessed by a second anesthesiologist who was unaware of the groups. Bispectral index values(until reach to 95), heart rate, blood pressure, ETco2 and oxygen saturation were determined in all the patients before administration and every one minute after injection of the test drug for 30 minutes. The following variables were measured in both groups: Time to eye opening in response to vocal request, time to extubation and response to command (hand squeezing) after injection of aminophyline or placebo. The Aldrete scores (until reach to 9) were recorded on arrival to the postanesthesia care unit (PACU) and every 5 minutes in the first 20 minutes and then every 10 minutes for one hour in the PACU. 

***Statistics: ***Data are expressed as mean±SD. Analysis of demographic values were performed by the T-test and one way analysis of variance. Analysis of bispectral index values, HR, BP were performed by a t-test for independent groups, arithmetic calculations and Aldrete scores were performed by the t-test. (β=2, α=.05, SD=1.7)

Statistical analysis was performed with SPSS 15, all graphs were made with excel Microsoft software. All data are presented as mean (SD) or mean [95% CI]. For analysis, the main study variables: BIS, heart rate and blood pressure were normalized to zero at the time of injection of the study medication to eliminate the differences in absolute values between the study subjects since the study was designed to study changes in variables irrespective of the absolute baseline value. A probability value < 0.05 was considered to be statistically significant.

## RESULTS

**Fig.1:**
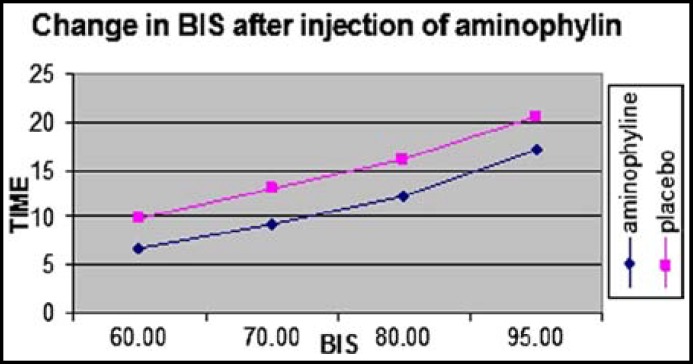
Change in BIS after injection of aminophylline.

**Fig.2:**
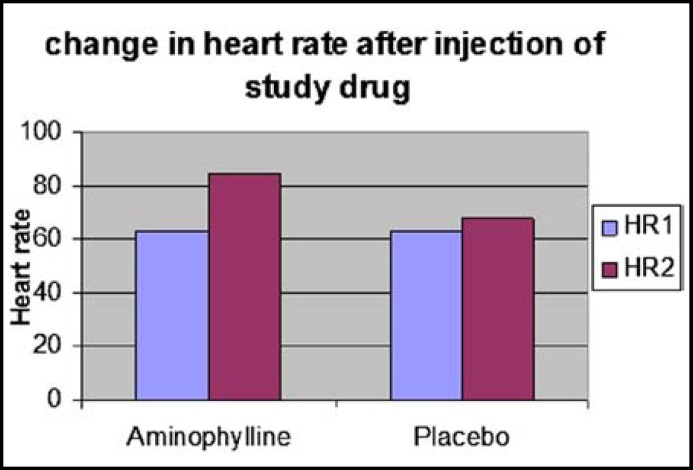
Change in heart rate after injection of study drug.

**Fig.3:**
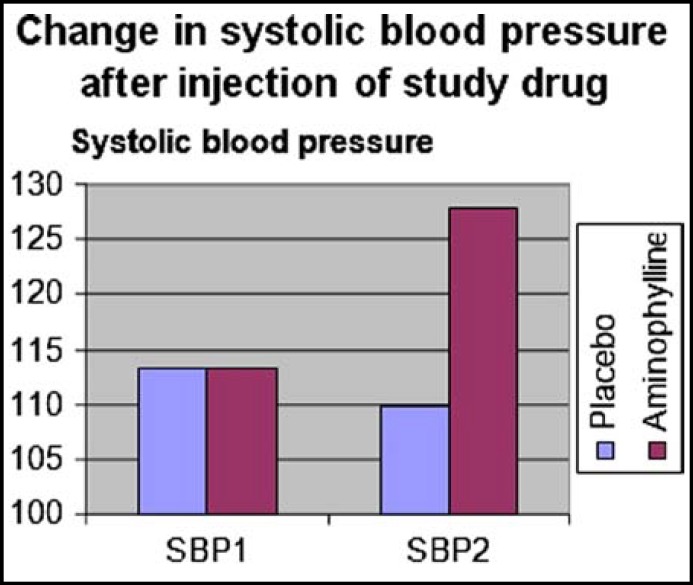
Change in systolic blood pressure after injection of study drug.

Patients in the two groups were comparable with respect to age, body weight, ASA status and duration of surgery (Table-I). ETCO2, SpO2 and ECG values were similar in the two groups and comparable to pre injection values (Table-II). There was no statistically significant difference in the BIS scores between two groups prior to the injection of the test drug (p>0.05). After injection of the test drug, BIS scores were found to be significantly higher (p<0.001) in Group A at 1 to 25 min (Fig.1). Heart rate and blood pressure were found to be higher significantly after injection of aminophylline compared with the placebo group (p<0.001) (Fig. 2 and 3). Recovery times in all measured variables (time to eye opening, extubation, hand grip and awake time) were significantly shorter in Group A (p<0.001) (Table-III). All of the patients had Aldrete scores 9 in the postanesthesia care unit less than one hour after termination of operation.

## DISCUSSION

The main result of our study was that aminophylline led to improvement in early recovery parameters and BIS values after general anesthesia. Overall, mechanism of action of drugs thought to be change in cellular systems, ion channels, secondary messengers and neurotransmitters. Change in adenosine function may be one of the mechanisms of action for some anesthetics. Four subtypes of adenosine receptors are expressed in the CNS: A1, A2A, A2B and A3. A1 and A2A receptors modulate cortical ACh release, behavioral arousal, and sleep. The mechanism for the antihypnotic effect of aminophylline is thought to be suppression of adenosine receptors in the CNS.^19^^,^^20^ Meanwhile, some authors have reported that caffeine which is structurally similar to aminophylline, decreases GABA-ergic neurotransmission.^21^

**Table-I:** Demographic variables between two groups

*Qualitative Variable*	*Group P*	*Group A*
***Male***	***33***	***35***
***Female***	***13***	***11***
***Age***	***31.9±6.7***	***32.2±6.8***
***Weight***	***65.6±10.3***	***65.5±8.8***

**Table-II:** Quantitative variables between two groups

*Variables*	*SPO2(1)*	*SPO2(2)*	*ETCO2*
***Group A***	***97±0.1***	***96.9±0.1***	***34.1±0.1***
***Group P***	***97.1±0.1***	***97±0.1***	***33.9±0.1***

**SPO2(1):Oxygen Saturation before injection of study drug.**

**SPO2(2):Oxygen Saturation after injection of study drug.**

**Table-III:** Recovery variables of two groups

*Variables*	**Awake time**	*Time to hand grip*	*Time to eye opening*	*Extubation time*
Group				
***Group A***	***18±3.5***	***20.8±3.5***	***20±3.7***	***21.4±3.7***
***Group P***	***24.1±2.4***	***23.9±2.1***	***22.6±1.9***	***23.9±2***
p-value	.001	.001	.001	.001
95% confidence interval of the difference	Lower: -7.31683	Lower: -4.31991	Lower: -3.81988	Lower: -3.77154
	Upper: -4.81361	Upper: -1.89748	Upper: -1.35403	Upper: -1.27194

SBP1: Systolic blood pressure before injection of study drug.

SBP2: Systolic blood pressure after injection of study drug.

In recent years BIS has been used for measuring the hypnotic component of anesthesia. In our study we used propofol for anesthesia maintenance and remifentanil which is an ultrashort acting opioid and has no effect on BIS. On the other hand, we didn’t use any muscle relaxation with the idea not to interfere with BIS values. In this study BIS values were only under effect of propofol and aminophylline. Aminophylline has been known to decrease the sedative and hypnotic effect of barbiturates.^16^ This study showed the anti-hypnotic effect of aminophylline on propofol since we avoided factors that cause incorrect BIS data like other drugs and electric device interference.^22^ Moreover, we selected a brief surgery (herniorrhaphy), which lasted no more than one hour, then our subjects were homogenous because we wanted to avoid accumulating effect of anesthetics. On the other hand, one of our concerns was pro-convulsant activity of aminophylline that artifactually may have contributed to the higher BIS scores in the aminophylline group. We never observed clinical epileptiform activity.

Our results concur with previous reports. Similar observations during recovery time have been made, and support the hypothesis that aminophylline is partially able to antagonise the hypnotic and sedative effects of general anaesthetics. Turan et al.^10^^,^^12^ demonstrated that aminophylline shortens recovery times and improves cognitive functions following sevoflurane anesthesia and improve sevoflurane recovery time. M. Hupfl, et al.^11^ demonstrated the effects of aminophylline on bispectral index during inhalational and total intravenous anesthesia which was associated with significant increase in BIS up to 10 min after aminophylline injection.

Our study showed that aminophylline can be used in outpatient surgery safely. Although we observed an increase in heart rate and blood pressure in our subjects, they didn’t need any intervention. Meanwhile, theophylline metabolism occurs in the liver by cytochrome P450 system, then caution needs to be exercised when using other drugs that are also metabolized by the cytochrome system. Conditions such as hepatic dysfunction and heart failure can reduce the elimination of theophylline; and low albumin states reduces the amount of protein-bound drug in the blood, then in these situations, aminophylline should be used with caution. When using aminophylline in recovery of anesthesia, these effects must be evaluated carefully and precautions must be taken in the speed of the injection. 

In conclusion, we have shown that aminophylline improves the early clinical recovery after TIVA (propofol+remifentanil) and this correlates with higher BIS values when compared with placebo.

## Authors Contribution:

**Ghaffaripour, Mahmoudi, Rahimi, **conceived, designed and did statistical analysis & editing of manuscript.

**Rahimi, Kazemi, Sahmeddini, Khosravi **did data collection and manuscript writing.

**Ghaffaripour, Chohedri** did review and final approval of manuscript.

**Ghaffaripour** takes the responsibility and is accountable for all aspects of the work in ensuring that questions related to the accuracy or integrity of any part of the work are appropriately investigated and resolved.

## Notes

***Source of funding:*** Deputy of research, Shiraz University of Medical Sciences.
